# Supplementary material for: Functional characterization of a single nucleotide polymorphism associated with Alzheimer’s disease in a hiPSC-based neuron model
Source: PLoS One. 2023 Sep 26;18(9):e0291029. doi: 10.1371/journal.pone.0291029 (PMC10521995; doi:10.1371/journal.pone.0291029)
Supplement: S1 Fig — Finngen metadata (data freeze 3) for rs148726219 showing associations with Alzheimer’s disease and dementia. (PDF) [file pone.0291029.s001.pdf]

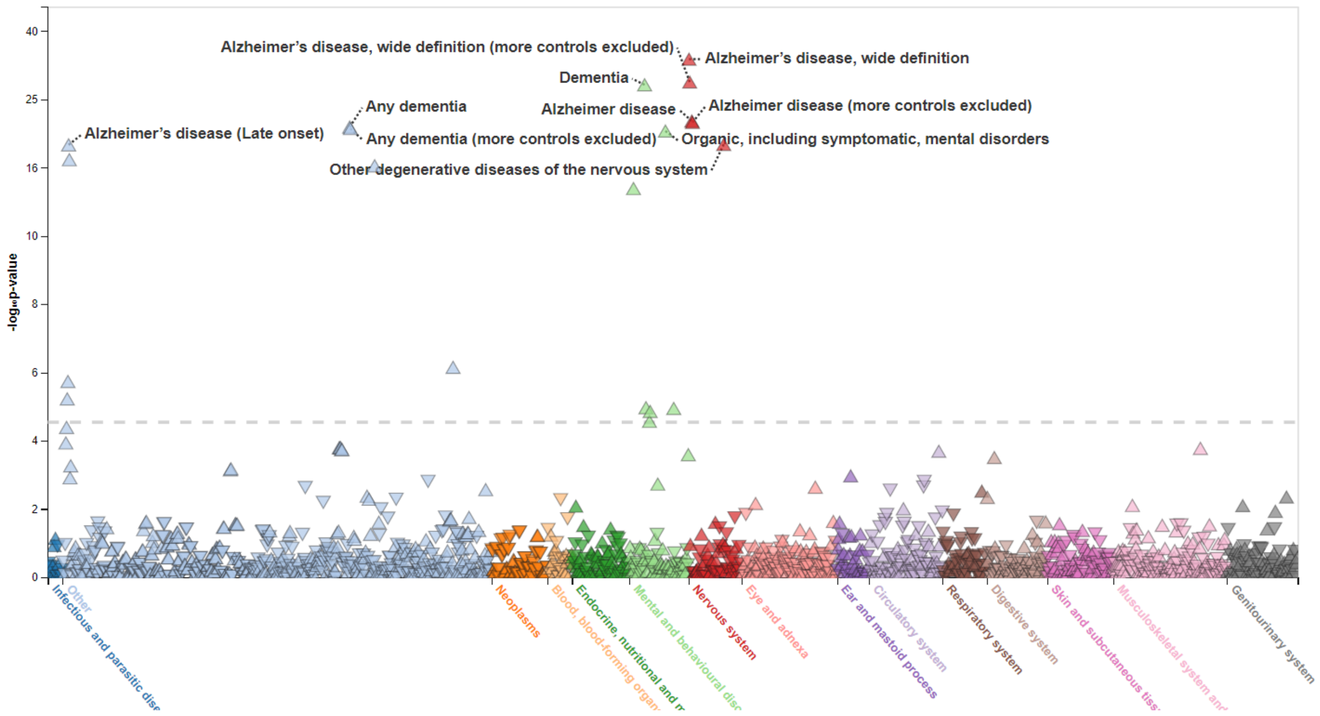

Supplemental Figure 1. Finngen disease associations for rs148726219.
Finngen metadata (data freeze 3) for rs148726219 showing associations with Alzheimer's disease and dementia.
