# Supplementary material for: Functional characterization of a single nucleotide polymorphism associated with Alzheimer’s disease in a hiPSC-based neuron model
Source: PLoS One. 2023 Sep 26;18(9):e0291029. doi: 10.1371/journal.pone.0291029 (PMC10521995; doi:10.1371/journal.pone.0291029)
Supplement: S2 Fig — G-banding was performed on 20 metaphase cells. All lines exhibited normal male karyotypes. A. WT-2A1. B. Het-2D2. C. Het-2G6. D. Hom-2B11. E. Hom-2H6. (PDF) [file pone.0291029.s002.pdf]

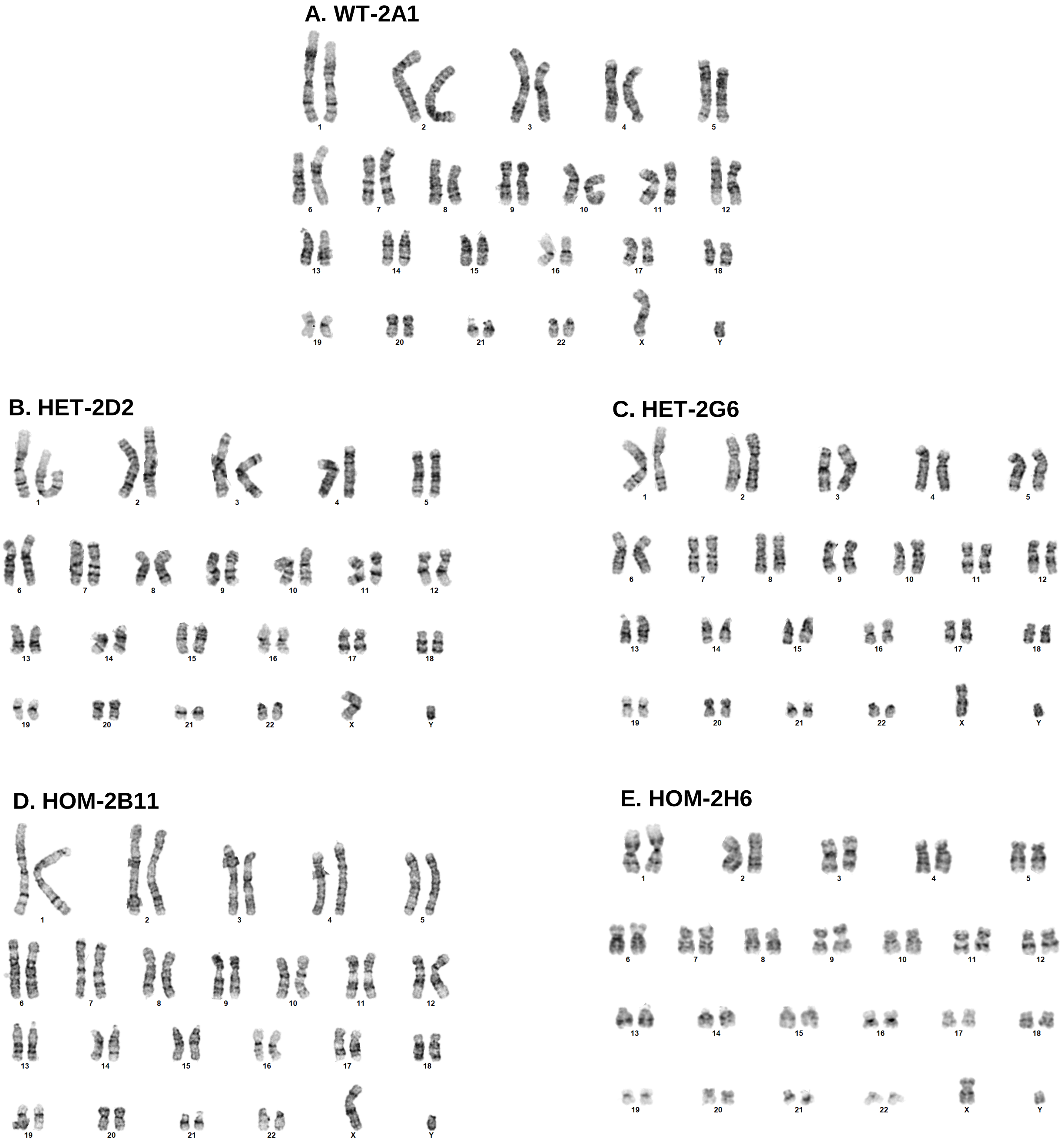

Supplemental Figure 2. Cytogenetic analysis of rs148726219-edited hiPSC clones. G-banding was performed on 20 metaphase cells. All lines exhibited normal male karyotypes. **A.** WT-2A1. **B.** Het-2D2. **C.** Het-2G6. **D.** Hom-2B11. **E.** Hom-2H6.
