# Supplementary material for: Functional characterization of a single nucleotide polymorphism associated with Alzheimer’s disease in a hiPSC-based neuron model
Source: PLoS One. 2023 Sep 26;18(9):e0291029. doi: 10.1371/journal.pone.0291029 (PMC10521995; doi:10.1371/journal.pone.0291029)
Supplement: S3 Fig — A. Representative immunofluorescence images of CUX1 (green), SOX2 (orange), and TUJ1 (red) are shown for the BIONi010-C-13 parental line, and WT-2A1, HET-2D2, HET-2G6, HOM-2B11, and HOM-2H6 clones. The merged images includes DAPI nuclear counterstain (blue). Scale bars = 100 um. B. Relative cell viability of each iN line at day 23 measured by Cell Titer Glo. Relative fluorescence units from 3 independent differentiation experiments, each with 6 technical replicates, was normalized to the WT-parental line (n = 3, n = 2 for HOM-2H6). ns = not significant. *p < 0.05 by one-way ANOVA with multiple comparisons. (PDF) [file pone.0291029.s003.pdf]

A.

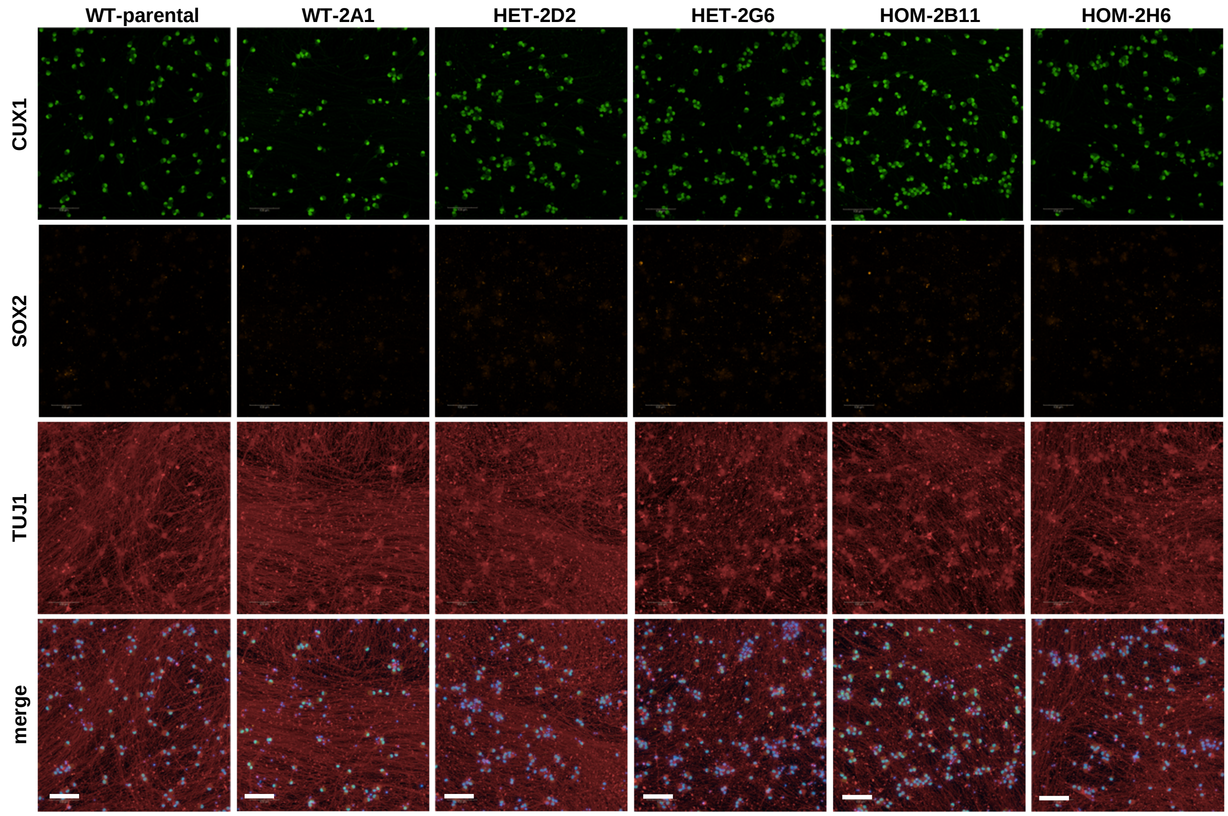

B.

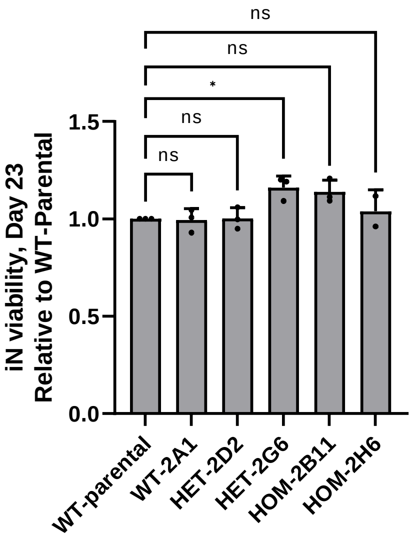

Supplemental Figure 3. Day 23 iNeurons do not show differences between clones in cell morphology, levels of neuronal marker proteins, or viability.

A. Representative immunofluorescence images of CUX1 (green), SOX2 (orange), and TUJ1 (red) are shown for the BIONi010-C-13 parental line, and WT-2A1, HET-2D2, HET-2G6, HOM-2B11, and HOM-2H6 clones. The merged images includes DAPI nuclear counterstain (blue). Scale bars = 100 um. **B.** Relative cell viability of each iN line at day 23 measured by Cell Titer Glo. Relative fluorescence units from 3 independent differentiation experiments, each with 6 technical replicates, was normalized to the WT-parental line (n=3, n=2 for HOM-2H6). ns = not significant. *p < 0.05 by one-way ANOVA with multiple comparisons.
