# Supplementary material for: Functional characterization of a single nucleotide polymorphism associated with Alzheimer’s disease in a hiPSC-based neuron model
Source: PLoS One. 2023 Sep 26;18(9):e0291029. doi: 10.1371/journal.pone.0291029 (PMC10521995; doi:10.1371/journal.pone.0291029)
Supplement: S4 Fig — A. Principal component analysis (PCA) of high-throughput qPCR of BIONi010-C-13 parental, WT-2A1, HET-2D2, HET-2G6, HOM-2B11, and HOM-2H6 lines at days 0, 2, 6, 13, and 23 of 1 representative iNeuron differentiation. Three technical replicates of each line are shown per timepoint. Samples cluster by timepoint, not by genotype or clone. NTC = qPCR no template control; UHRR = universal human reference RNA used as a positive control for gene expression. B-C. Neurogenin2 (NEUROG2, NGN2) expression measured by high-throughput qPCR as in (A). Values were normalized relative to the geometric mean of 3 housekeeping genes (GAPDH, EIF4A2, RPL13) and expressed as fold change values (DCt). Data grouped by timepoint (B) and by line (C) illustrates increased NEUROG2 expression with DOX induction (days 2 and 6), and reduced expression following treatment (days 13 and 23). No expression is detected at day 0, indicating no inherent leakiness of the Tet operator. (PDF) [file pone.0291029.s004.pdf]

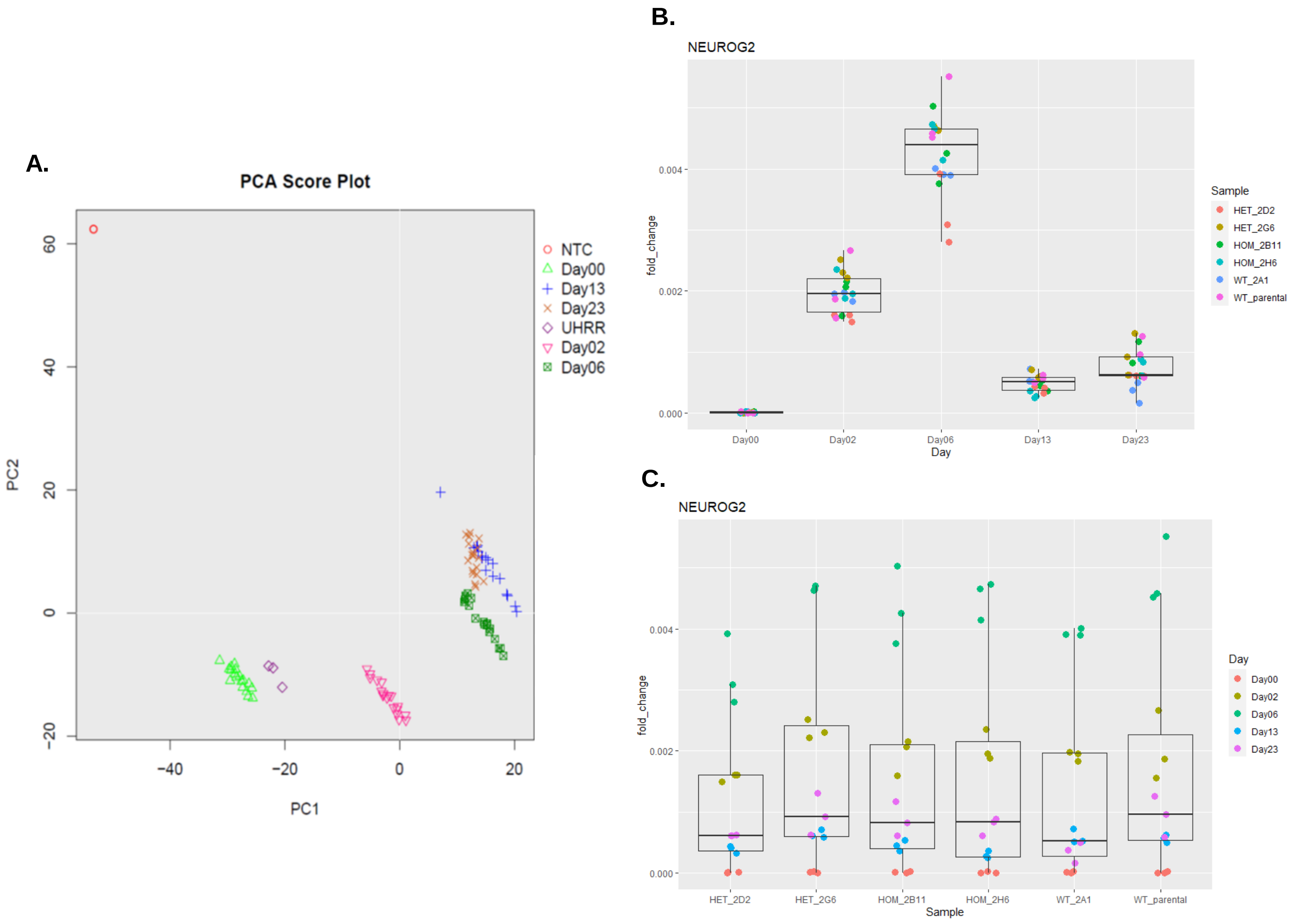

Supplemental Figure 4. High-throughput qPCR data from rs148726219-edited lines.
A. Principal component analysis (PCA) of high-throughput qPCR of BIONi010-C-13 parental, WT-2A1, HET-2D2, HET-2G6, HOM-2B11, and HOM-2H6 lines at days 0, 2, 6, 13, and 23 of 1 representative iNeuron differentiation. Three technical replicates of each line are shown per timepoint. Samples cluster by timepoint, not by genotype or clone. NTC = qPCR no template control; UHRR = universal human reference RNA used as a positive control for gene expression. **B-C.** Neurogenin2 (*NEUROG2, NGN2*) expression measured by high-throughput qPCR as in (A). Values were normalized relative to the geometric mean of 3 housekeeping genes (*GAPDH, EIF4A2, RPL13*) and expressed as fold change values (ΔCt). Data grouped by timepoint (**B**) and by line (**C**) illustrates increased *NEUROG2* expression with DOX induction (days 2 and 6), and reduced expression following treatment (days 13 and 23). No expression is detected at day 0, indicating no inherent leakiness of the Tet operator.
