# Supplementary material for: Functional characterization of a single nucleotide polymorphism associated with Alzheimer’s disease in a hiPSC-based neuron model
Source: PLoS One. 2023 Sep 26;18(9):e0291029. doi: 10.1371/journal.pone.0291029 (PMC10521995; doi:10.1371/journal.pone.0291029)
Supplement: S5 Fig — Expression of genes related to A. mature neurons (DCX, DLG4, MAP2, MAPT, RBFOX3, SLC17A6, SLC17A7, SYN1, SYP, TUBB3), B. pluripotency (LIN28A, MKI67, NANOG, POU5F1), C. neural progenitor cells (DACH1, OTX2, PAX6, PROM1, SOX2), D. non-neuronal cells (CDX2, GATA4, GFAP, OLIG1, S100B, SLC1A3, SOX17, TBXT), and E. AD-associated markers (APOE, 4R isoform of MAPT, PSEN1, APP). Transcripts were measured by high-throughput qPCR of BIONi010-C-13 parental, WT-2A1, HET-2D2, HET-2G6, HOM-2B11, and HOM-2H6 lines at days 0, 2, 6, 13, and 23 of 1 representative iN differentiation. Values were normalized relative to the geometric mean of 3 housekeeping genes (GAPDH, EIF4A2, RPL13) and expressed as fold change values. Three technical replicates of each line are shown per timepoint. (PDF) [file pone.0291029.s005.pdf]

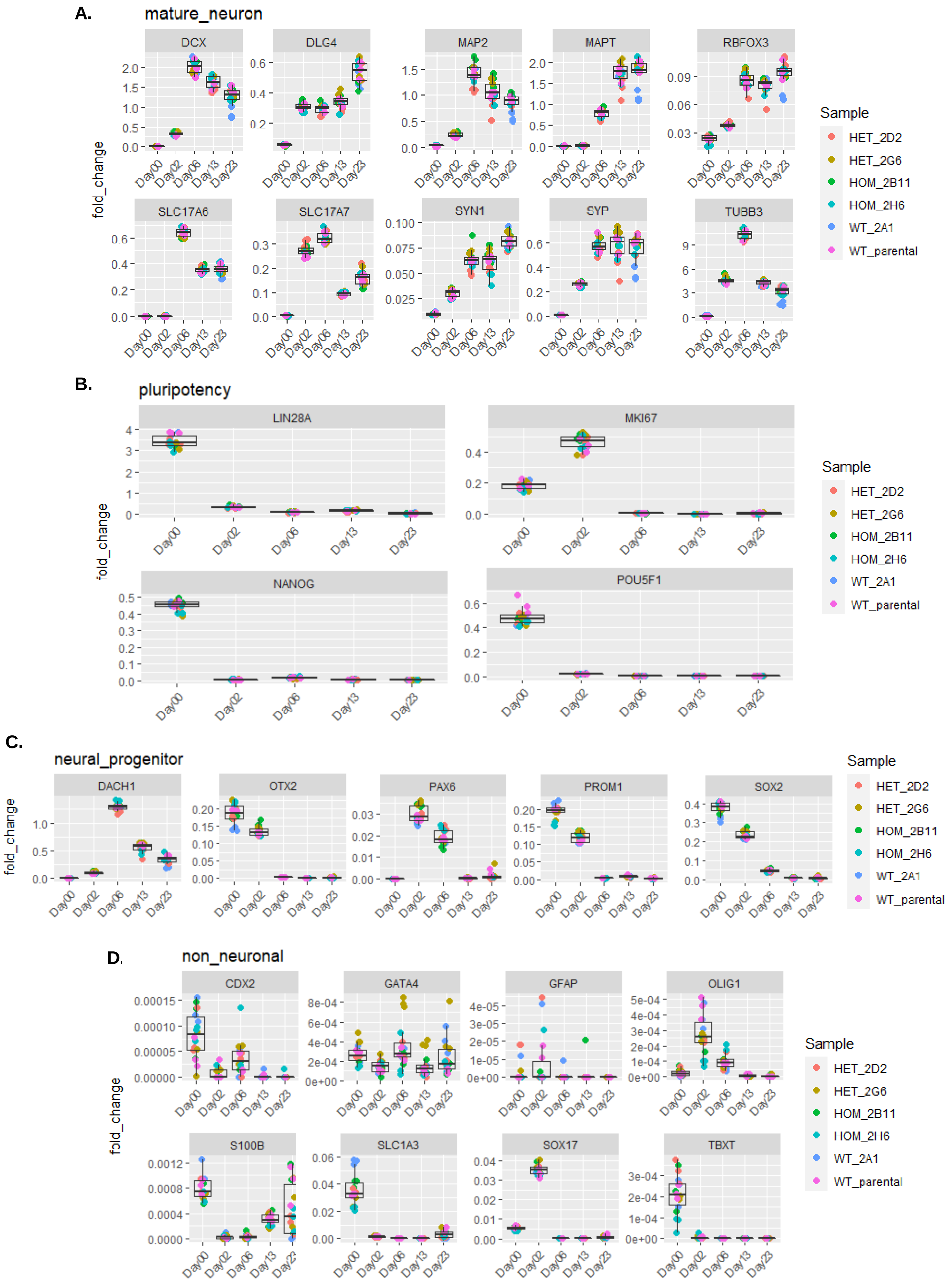

Supplemental Figure 5. Legend on next page.

E.

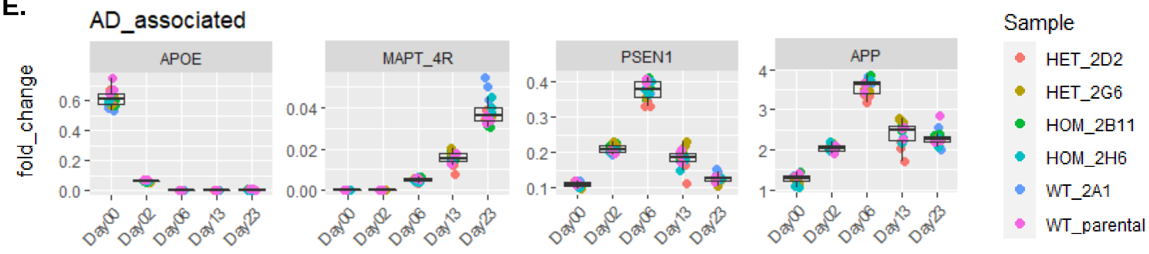

Supplemental Figure 5 (continued). High-throughput qPCR data of marker genes from rs148726219-edited lines.
Expression of genes related to **A.** mature neurons (*DCX, DLG4, MAP2, MAPT, RBFOX3, SLC17A6, SLC17A7, SYN1, SYP, TUBB3)*, **B.** pluripotency (*LIN28A, MKI67, NANOG, POU5F1),* **C.** neural progenitor cells (*DACH1, OTX2, PAX6, PROM1, SOX2*), **D.** non-neuronal cells *(CDX2, GATA4, GFAP, OLIG1, S100B, SLC1A3, SOX17, TBXT),* and **E.** AD-associated markers (*APOE, 4R* isoform of *MAPT, PSEN1, APP).* Transcripts were measured by high-throughput qPCR of BIONi010-C-13 parental, WT-2A1, HET-2D2, HET-2G6, HOM-2B11, and HOM-2H6 lines at days 0, 2, 6, 13, and 23 of 1 representative iN differentiation. Values were normalized relative to the geometric mean of 3 housekeeping genes (*GAPDH, EIF4A2, RPL13*) and expressed as fold change values. Three technical replicates of each line are shown per timepoint.
