# Supplementary material for: Functional characterization of a single nucleotide polymorphism associated with Alzheimer’s disease in a hiPSC-based neuron model
Source: PLoS One. 2023 Sep 26;18(9):e0291029. doi: 10.1371/journal.pone.0291029 (PMC10521995; doi:10.1371/journal.pone.0291029)
Supplement: S6 Fig — Expression of Aβ protein isoforms Aβ40 (A), Aβ38 (B), and Aβ42 (C) measured by MSD ELISA from media conditioned by BIONi010-C-13 parental, WT-2A1, HET-2D2, HET-2G6, HOM-2B11, and HOM-2H6 lines on day 23 of iN differentiation. The ratio of Aβ42/Aβ40 is also shown (D). Cells were untreated (blue bars), treated with vehicle control (DMSO; grey bars), or treated with DAPT to inhibit Aβ production (white bars) for 24 hrs prior to media collection. Values were normalized against a standard curve and expressed relative to total cell number, quantified as relative fluorescence units from Cell Titer Glo assays. n = 3 independent hiPSC-iNeuron differentiations (or n = 2 for HOM-2H6), with each represented by a black dot. No apparent differences in Aβ levels are observed between lines with heterozygous/homozygous alleles for rs148726219 and wild type lines. (PDF) [file pone.0291029.s006.pdf]

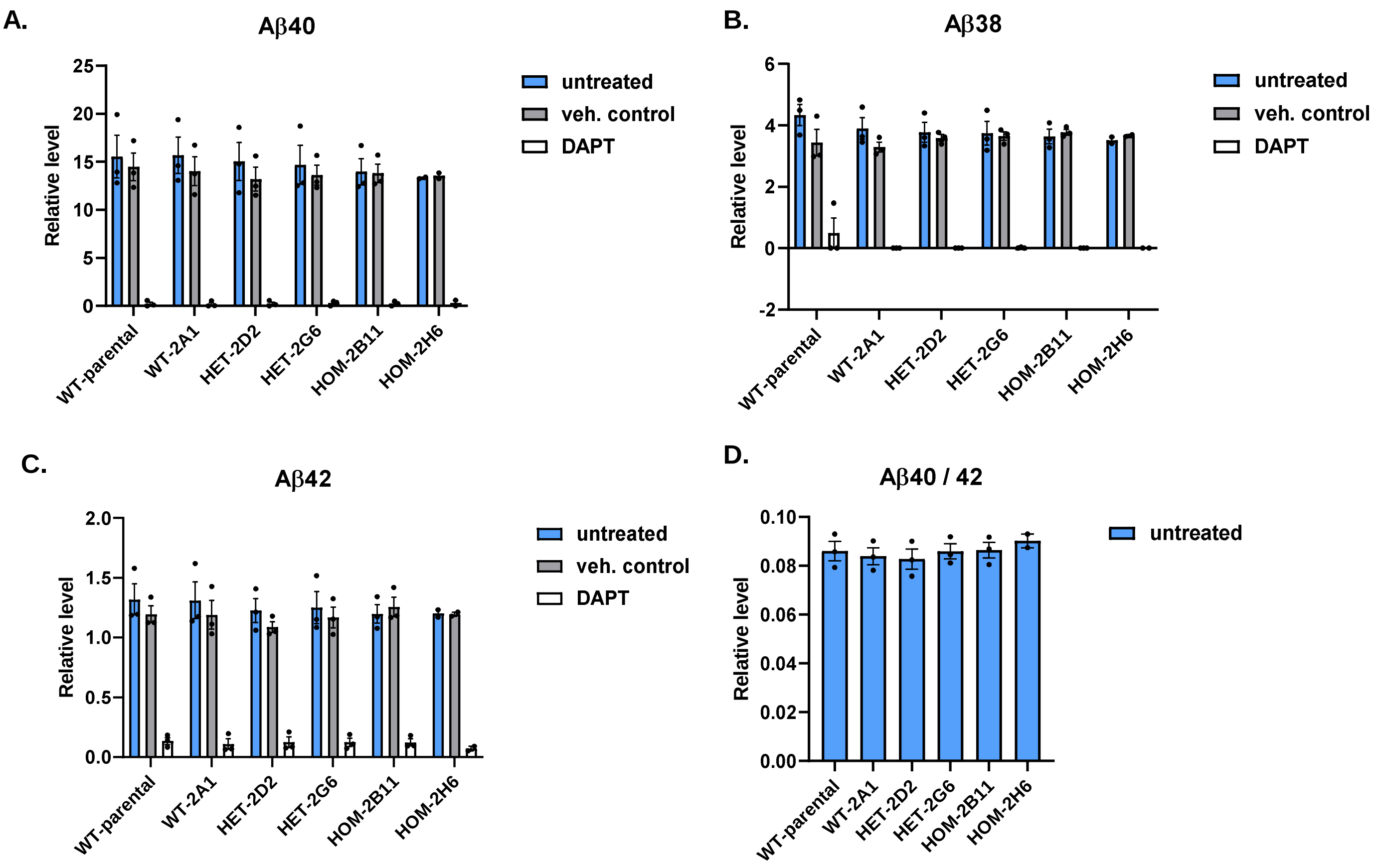

Supplemental Figure 6. Secreted levels of amyloid beta from rs148726219-edited lines.
Expression of Aβ protein isoforms Aβ40 (**A**), Aβ38 (**B**), and Aβ42 (**C**) measured by MSD ELISA from media conditioned by BIONi010-C-13 parental, WT-2A1, HET-2D2, HET-2G6, HOM-2B11, and HOM-2H6 lines on day 23 of iN differentiation. The ratio of Aβ42/Aβ40 is also shown (**D**). Cells were untreated (blue bars), treated with vehicle control (DMSO; grey bars), or treated with DAPT to inhibit Aβ production (white bars) for 24 hrs prior to media collection. Values were normalized against a standard curve and expressed relative to total cell number, quantified as relative fluorescence units from Cell Titer Glo assays. n = 3 independent hiPSC-iNeuron differentiations (or n = 2 for HOM-2H6), with each represented by a black dot. No apparent differences in Aβ levels are observed between lines with heterozygous/homozygous alleles for rs148726219 and wild type lines.
