# Supplementary material for: Functional characterization of a single nucleotide polymorphism associated with Alzheimer’s disease in a hiPSC-based neuron model
Source: PLoS One. 2023 Sep 26;18(9):e0291029. doi: 10.1371/journal.pone.0291029 (PMC10521995; doi:10.1371/journal.pone.0291029)
Supplement: S7 Fig — PCA plots are colored by time point (A), colored by rs148726219-edited clone (B), and colored by BIONi010-C-13 parental line versus WT-2A1 clone (C). Samples cluster by time point, not by line. No apparent differences are detectable between the parental line and WT clone. (PDF) [file pone.0291029.s007.pdf]

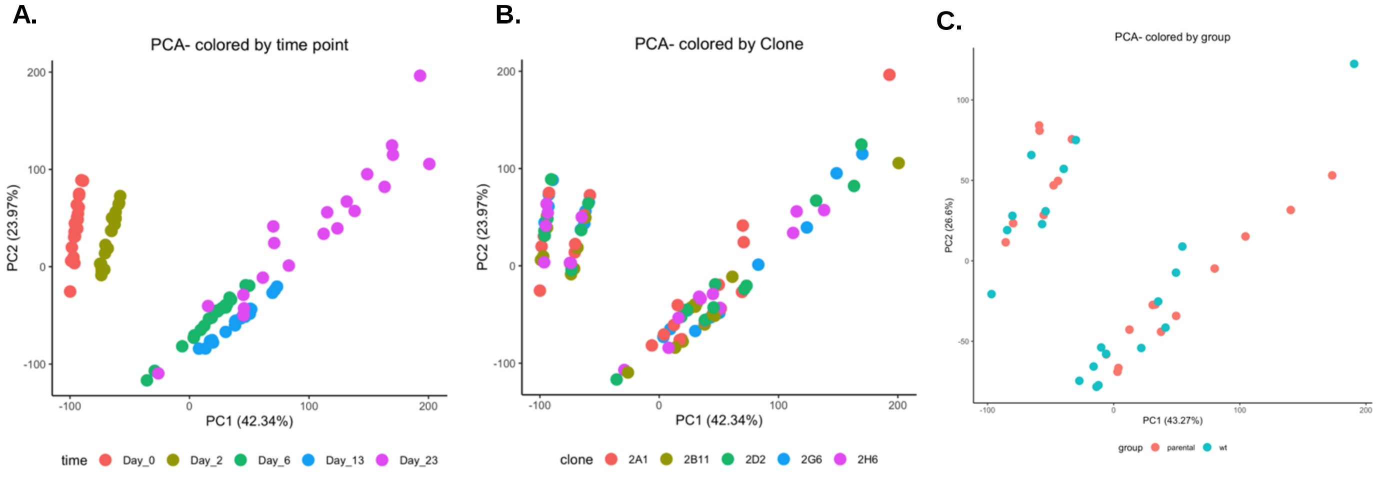

Supplementary Figure 7. Principle component analysis plots of RNA-seq data.
PCA plots are colored by time point (**A**), colored by rs148726219-edited clone (**B**), and colored by BIONi010-C-13 parental line versus WT-2A1 clone (**C**). Samples cluster by time point, not by line. No apparent differences are detectable between the parental line and WT clone.
