# Supplementary material for: Functional characterization of a single nucleotide polymorphism associated with Alzheimer’s disease in a hiPSC-based neuron model
Source: PLoS One. 2023 Sep 26;18(9):e0291029. doi: 10.1371/journal.pone.0291029 (PMC10521995; doi:10.1371/journal.pone.0291029)
Supplement: S9 Fig — Heterozygous clones (HET-2D2, HET-2G6) were compared to the WT clone (WT-2A1) at days 0, 2, 6, 13, and 23 of hiPSC-iNeuron differentiation. Each gene is represented by a dot showing–log10(adjusted p-value) (ie. FDR) and log(fold-change) (logFC) values. Downregulated genes in blue, upregulated genes in red. (PDF) [file pone.0291029.s009.pdf]

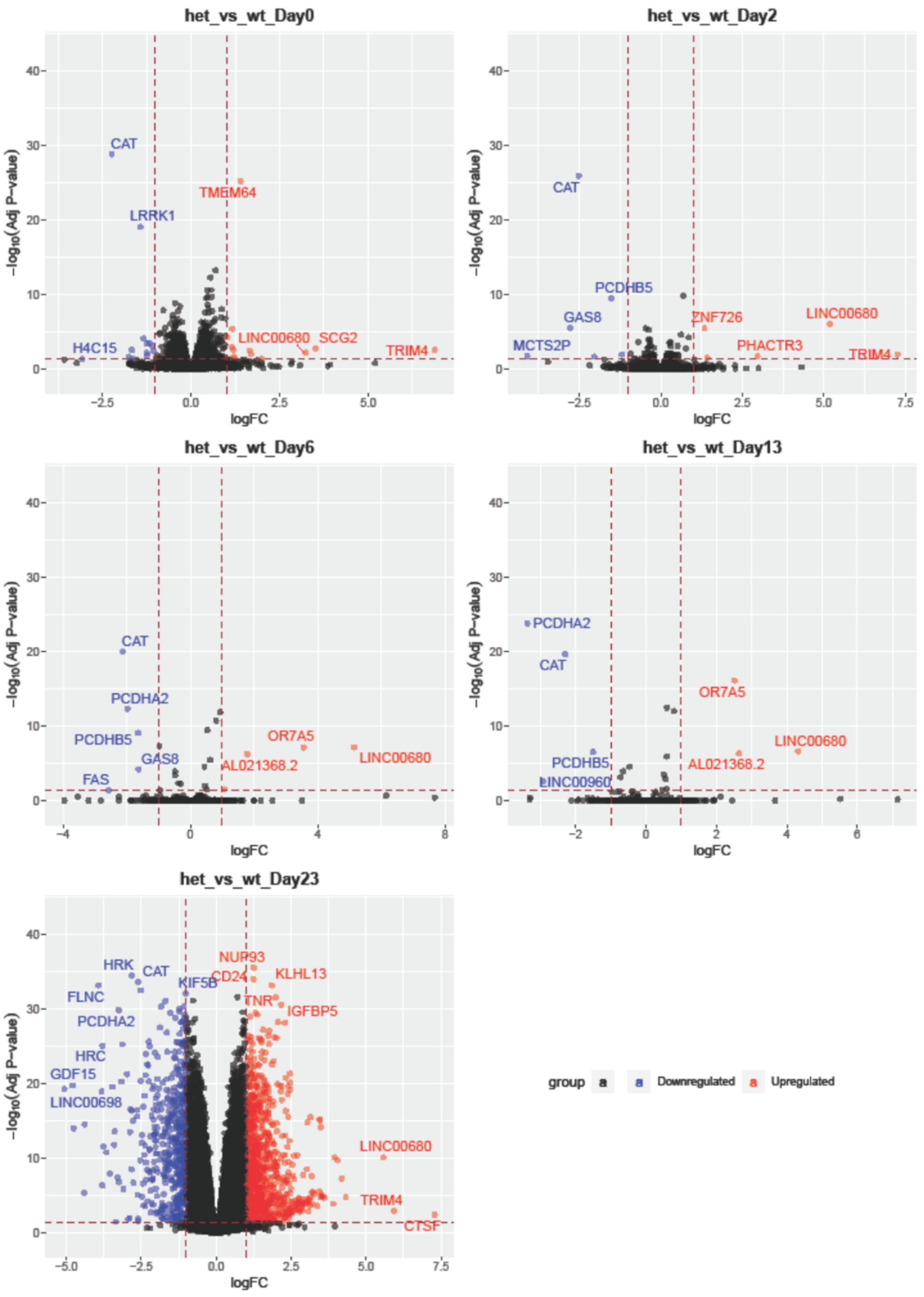

Supplementary Figure 9. Volcano plots of RNA-seq data from heterozygous clones versus wild type. Heterozygous clones (HET-2D2, HET-2G6) were compared to the WT clone (WT-2A1) at days 0, 2, 6, 13, and 23 of hiPSC-iNeuron differentiation. Each gene is represented by a dot showing –log10(adjusted p-value) (ie. FDR) and log(fold-change) (logFC) values. Downregulated genes in blue, upregulated genes in red.
