# Supplementary material for: Functional characterization of a single nucleotide polymorphism associated with Alzheimer’s disease in a hiPSC-based neuron model
Source: PLoS One. 2023 Sep 26;18(9):e0291029. doi: 10.1371/journal.pone.0291029 (PMC10521995; doi:10.1371/journal.pone.0291029)
Supplement: S10 Fig — Values for BIONi010-C-13 WT-parental line and WT-2A1 clone (grey bars), rs148726219-heterozygous clones (blue bars), and homozygous clones (green bars) day 0, 2, 6, 13, and 23 of hiPSC-iNeuron differentiation are shown. Cq values were normalized to the geometric mean of 3 housekeeping genes (B2M, ACTB, GAPDH) and where possible, are expressed relative to the WT-parental line. A. Total FOSB transcript. Day 0, n = 3; Day 2, n = 2; Day 23, n = 4. B. DFOSB. Day 0, n = 3; Day 2, n = 2; Day 23, n = 4. C. Total ERCC1 transcript. Day 0, n = 3; Day 2, n = 2; Day 23, n = 4. D. ERCC1 long transcript isoform. Day 0, n = 3; Day 2, n = 2; Day 23, n = 4. E. CAT. n = 2. F. PCDHB5. n = 2. (PDF) [file pone.0291029.s010.pdf]

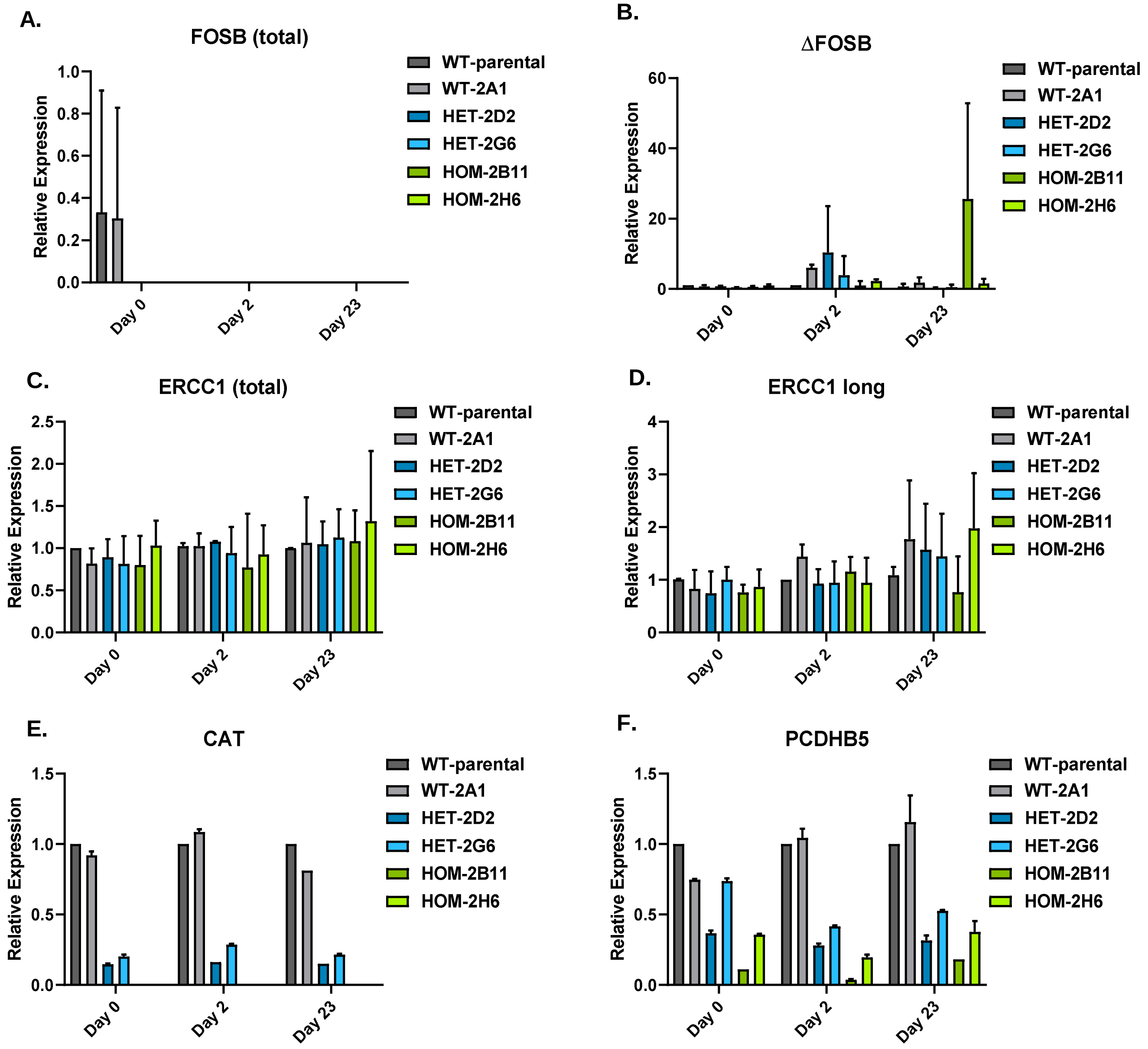

Supplementary Figure 10. Gene expression measured by SYBR Green RT-qPCR.
Values for BIONi010-C-13 WT-parental line and WT-2A1 clone (grey bars), rs148726219-heterozygous clones (blue bars), and homozygous clones (green bars) day 0, 2, 6, 13, and 23 of hiPSC-iNeuron differentiation are shown. Cq values were normalized to the geometric mean of 3 housekeeping genes (*B2M, ACTB, GAPDH*) and where possible, are expressed relative to the WT-parental line. **A.** Total *FOSB* transcript. Day 0, n=3; Day 2, n=2; Day 23, n=4. **B.** *ΔFOSB*. Day 0, n=3; Day 2, n=2; Day 23, n=4. **C.** Total *ERCC1* transcript. Day 0, n=3; Day 2, n=2; Day 23, n=4. **D.** *ERCC1* long transcript isoform. Day 0, n=3; Day 2, n=2; Day 23, n=4. **E.** *CAT*. n=2. **F.** *PCDHB5*. n=2.
