# Supplementary material for: Functional characterization of a single nucleotide polymorphism associated with Alzheimer’s disease in a hiPSC-based neuron model
Source: PLoS One. 2023 Sep 26;18(9):e0291029. doi: 10.1371/journal.pone.0291029 (PMC10521995; doi:10.1371/journal.pone.0291029)
Supplement: S11 Fig — A. Western blot of day 23 iNeuron lysates from BIONi010-C-13 parental, WT-2A1, HET-2D2, HET-2G6, HOM- 2B11, and HOM-2H6 lines either untreated (-) or stimulated for 4 hrs with PMA (+). Blot was probed with an antibody against FOSB, which detects canonical FOSB (top band, ~48 kD) and DFOSB (bottom band, ~38 kD). GAPDH is shown as the loading control. B. Quantification of the blot in (A) by pixel densitometry for canonical FOSB (dark grey area) and DFOSB (light grey area) relative to GAPDH. The height of each bar represents the total amount of FOSB (canonical FOSB + DFOSB). n = 1. (PDF) [file pone.0291029.s011.pdf]

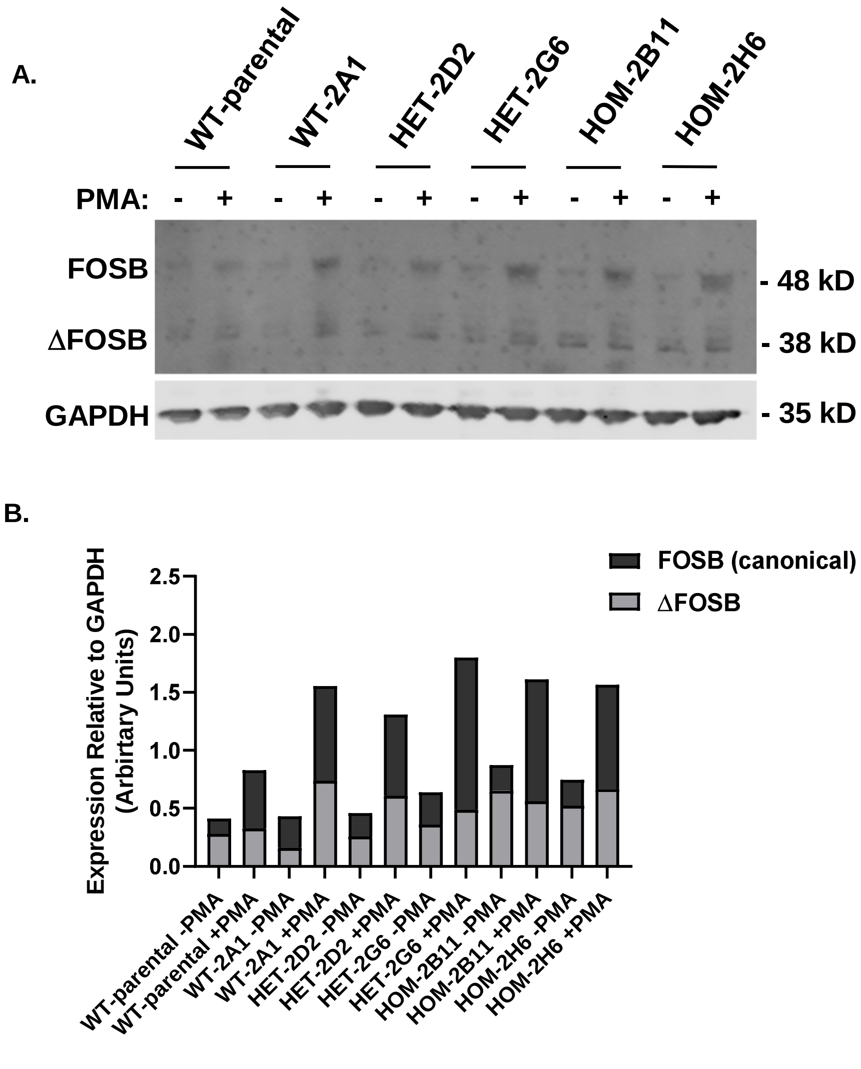

Supplementary Figure 11. Western blot for FOSB expression.
A. Western blot of day 23 iNeuron lysates from BIONi010-C-13 parental, WT-2A1, HET-2D2, HET-2G6, HOM-2B11, and HOM-2H6 lines either untreated (-) or stimulated for 4 hrs with PMA (+). Blot was probed with an antibody against FOSB, which detects canonical FOSB (top band, ~48 kD) and ΔFOSB (bottom band, ~38 kD). GAPDH is shown as the loading control. **B.** Quantification of the blot in (A) by pixel densitometry for canonical FOSB (dark grey area) and ΔFOSB (light grey area) relative to GAPDH. The height of each bar represents the total amount of FOSB (canonical FOSB + ΔFOSB). n=1.
