# Supplementary material for: Functional characterization of a single nucleotide polymorphism associated with Alzheimer’s disease in a hiPSC-based neuron model
Source: PLoS One. 2023 Sep 26;18(9):e0291029. doi: 10.1371/journal.pone.0291029 (PMC10521995; doi:10.1371/journal.pone.0291029)
Supplement: S12 Fig — Expression values are expressed as fragments per kilobase per million mapped reads (FPKM) for WT-2A1 (green), rs148726219-heterozygous (HET-2D2, HET-2G6; orange), and homozygous (HOM-2B11, HOM-2H6; purple) clones at day 0, 2, 6, 13, and 23 of hiPSC-iNeuron differentiation. Four technical replicates for each clone are shown with each represented by a black dot. A. ERCC1. B. BCL3. C. PPP1R37. D. RTN2. E. DMPK. F. APOE. (PDF) [file pone.0291029.s012.pdf]

A.

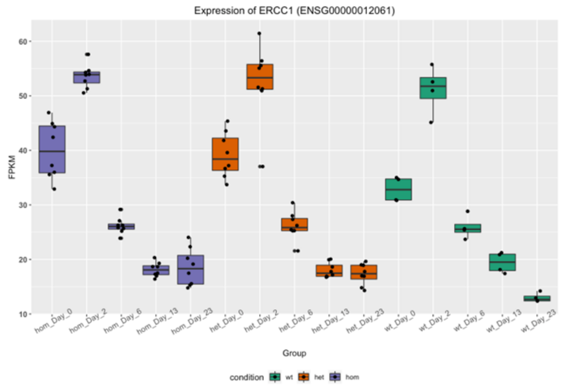

B.

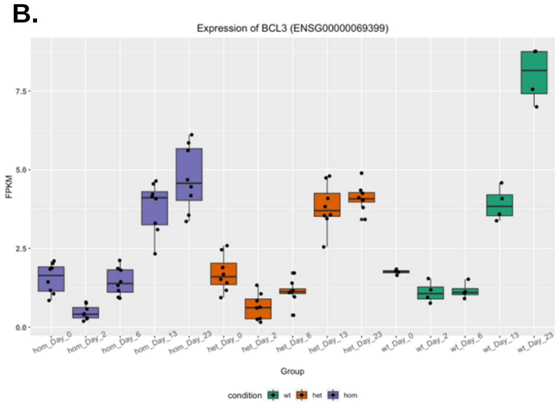

C.

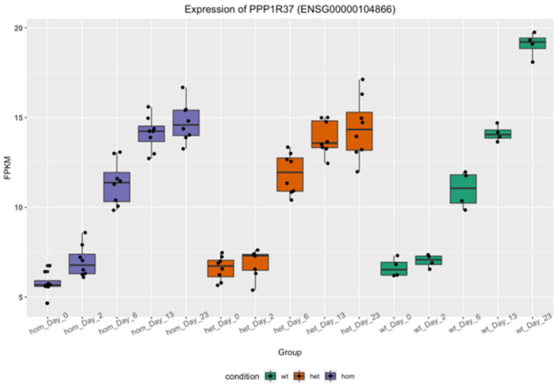

D.

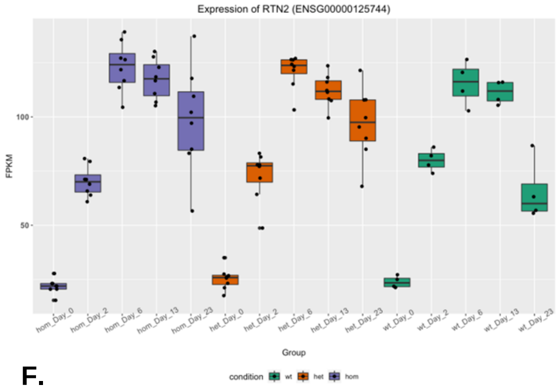

E.

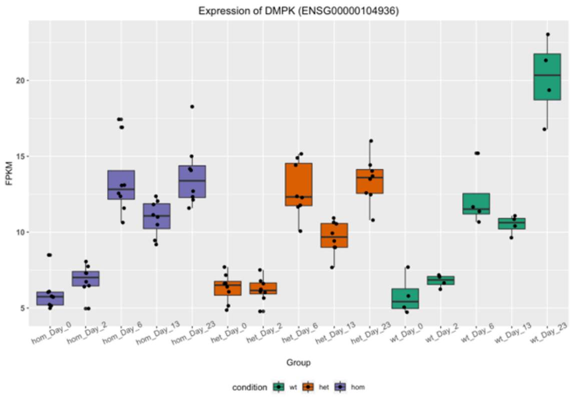

F.

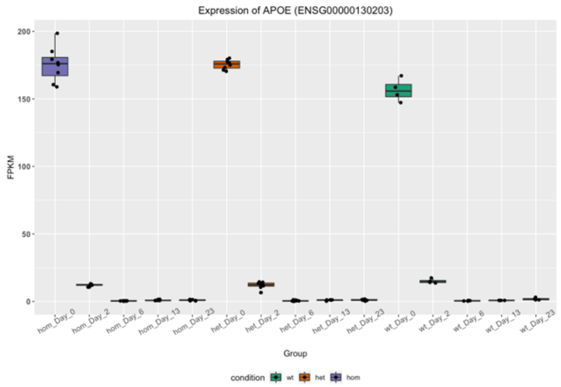

Supplementary Figure 12. Expression of genes within 1 Mb of rs148726219.
Expression values are expressed as fragments per kilobase per million mapped reads (FPKM) for WT-2A1 (green), rs148726219-heterozygous (HET-2D2, HET-2G6; orange), and homozygous (HOM-2B11, HOM-2H6; purple) clones at day 0, 2, 6, 13, and 23 of hiPSC-iNeuron differentiation. Four technical replicates for each clone are shown with each represented by a black dot. **A.** *ERCC1*. **B.** *BCL3*. **C.** PPP1R37. **D.** *RTN2*. **E.** *DMPK*. **F.** *APOE.*
