# Supplementary material for: Functional characterization of a single nucleotide polymorphism associated with Alzheimer’s disease in a hiPSC-based neuron model
Source: PLoS One. 2023 Sep 26;18(9):e0291029. doi: 10.1371/journal.pone.0291029 (PMC10521995; doi:10.1371/journal.pone.0291029)
Supplement: S13 Fig — A., B. Top 10 dysregulated pathways in homozygous clones (HOM-2B11, HOM-2H6; A) and heterozygous clones (HET-2D2, HET-2G6; B) compared to WT-2A1 clone.–Log(p-value) denotes level of significance for each pathway. Z-scores are denoted by numbers above each bar and colors (upregulated = orange, downregulated = navy). C. Top 20 dysregulated upstream regulators from comparison analysis between homozygous clones (left column) and heterozygous clones (right column) relative to WT-2A1 clones on day 23. Z-scores denote level of upregulation (orange) or downregulation (navy). (PDF) [file pone.0291029.s013.pdf]

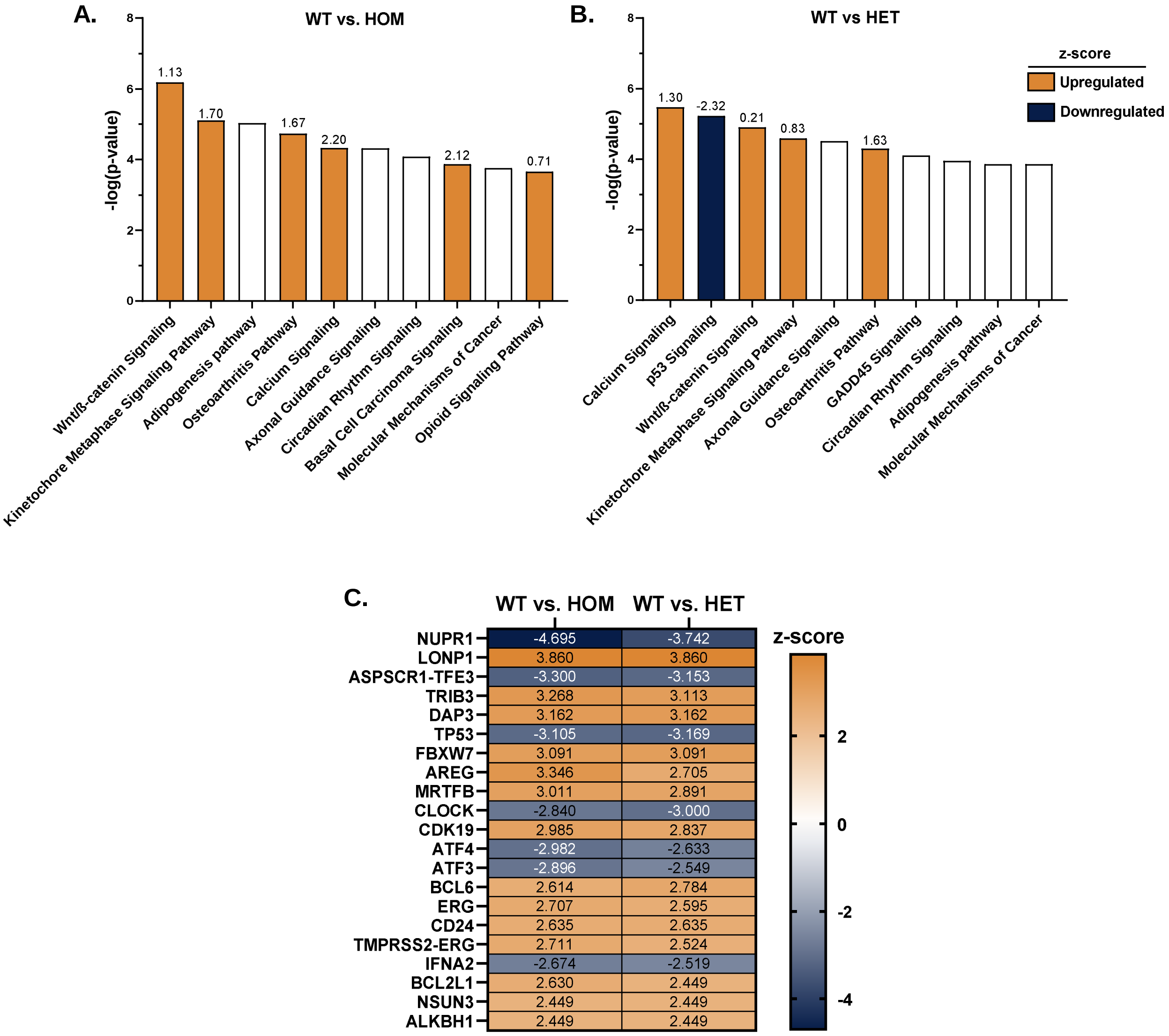

Supplementary Figure 13. IPA gene ontology analysis showing dysregulated pathways and upstream regulators in day 23 iNeurons.

A., B. Top 10 dysregulated pathways in homozygous clones (HOM-2B11, HOM-2H6; A) and heterozygous clones (HET-2D2, HET-2G6; B) compared to WT-2A1 clone. –Log(p-value) denotes level of significance for each pathway. Z-scores are denoted by numbers above each bar and colors (upregulated = orange, downregulated = navy). **C.** Top 20 dysregulated upstream regulators from comparison analysis between homozygous clones (left column) and heterozygous clones (right column) relative to WT-2A1 clones on day 23. Z-scores denote level of upregulation (orange) or downregulation (navy).
