# Supplementary material for: Functional characterization of a single nucleotide polymorphism associated with Alzheimer’s disease in a hiPSC-based neuron model
Source: PLoS One. 2023 Sep 26;18(9):e0291029. doi: 10.1371/journal.pone.0291029 (PMC10521995; doi:10.1371/journal.pone.0291029)
Supplement: S14 Fig — Differentially expressed genes (based on FDR < 0.05, logFC < -1.0 and > 1.0) are shown as heatmaps for each pathway. Comparison analysis between homozygous clones (left column) and heterozygous clones (right column) relative to WT-2A1 clones on day 23. Z-scores denote level of upregulation (orange) or downregulation (navy). Color denotes log fold change (logFC) values; orange = upregulated, navy = downregulated. (PDF) [file pone.0291029.s014.pdf]

RY8

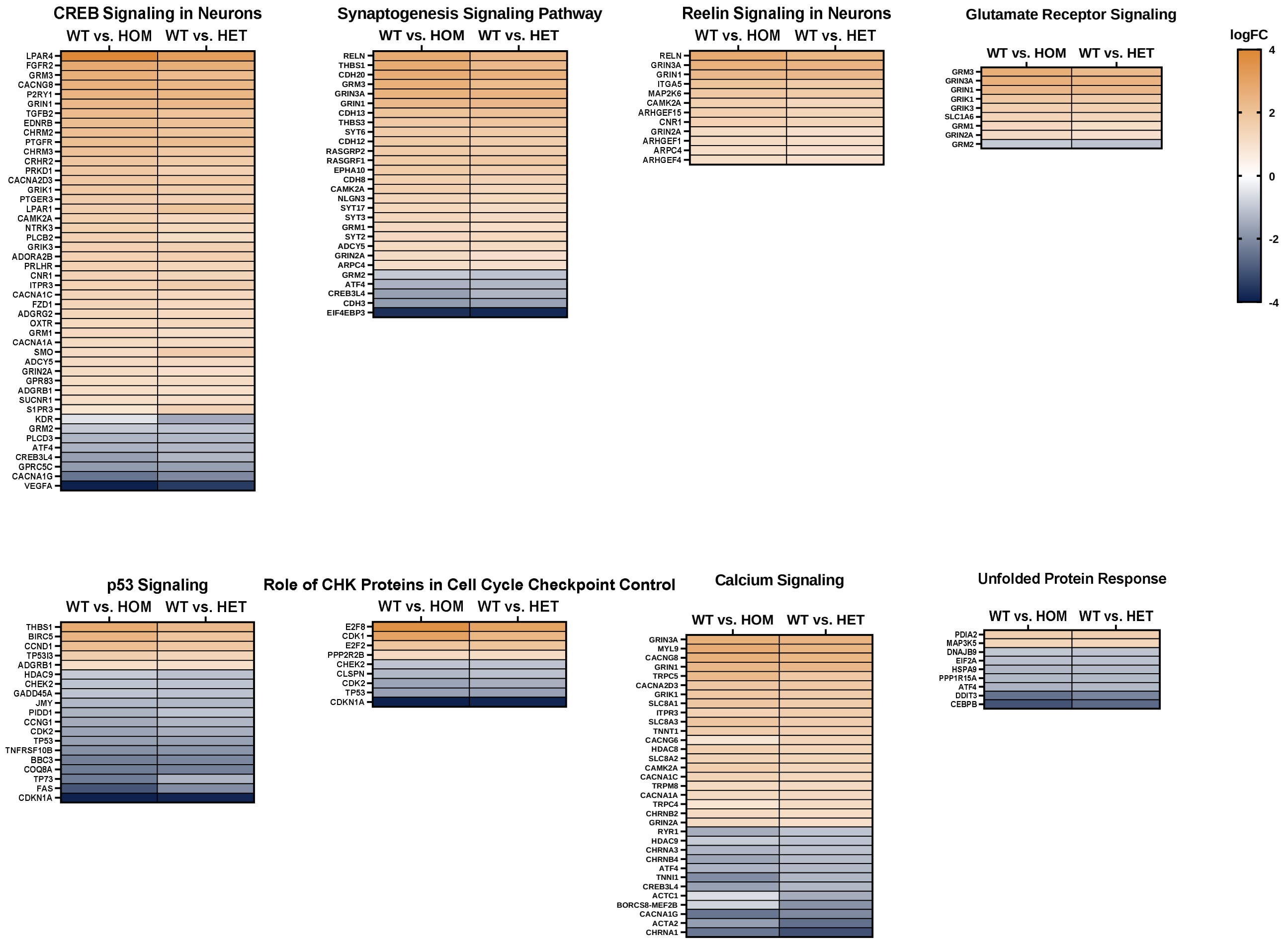

Supplementary Figure 14. IPA gene ontology analysis showing dysregulated genes within selected pathways at day 23.
Differentially expressed genes (based on FDR < 0.05, logFC < -1.0 and > 1.0) are shown as heatmaps for each pathway. Comparison analysis between homozygous clones (left column) and heterozygous clones (right column) relative to WT-2A1 clones on day 23. Z-scores denote level of upregulation (orange) or downregulation (navy). Color denotes log fold change (logFC) values; orange = upregulated, navy = downregulated.
