# Supplementary material for: Functional characterization of a single nucleotide polymorphism associated with Alzheimer’s disease in a hiPSC-based neuron model
Source: PLoS One. 2023 Sep 26;18(9):e0291029. doi: 10.1371/journal.pone.0291029 (PMC10521995; doi:10.1371/journal.pone.0291029)
Supplement: S16 Fig — Size of each node represents level of pathway enrichment. Fold change represents logFC values of each gene compared to the WT. A. Downregulated pathways. B. Upregulated pathways. (PDF) [file pone.0291029.s016.pdf]

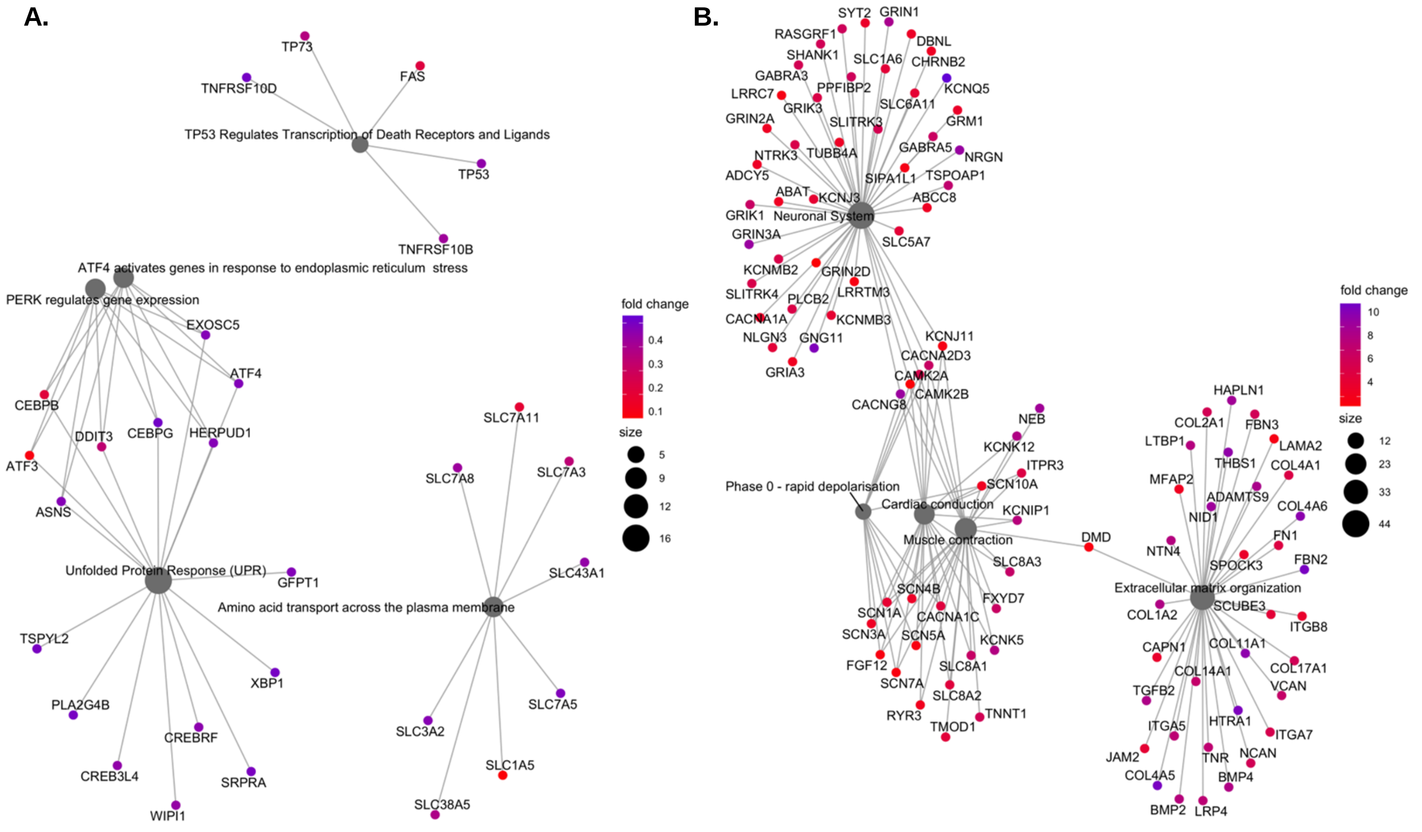


**Supplementary Figure 16. Cnetplots of rs148726219-homozygous iNeurons compared to the WT clone on day 23.**
Size of each node represents level of pathway enrichment. Fold change represents logFC values of each gene compared to the WT. **A.** Downregulated pathways. **B.** Upregulated pathways.
