# Supplementary material for: Functional characterization of a single nucleotide polymorphism associated with Alzheimer’s disease in a hiPSC-based neuron model
Source: PLoS One. 2023 Sep 26;18(9):e0291029. doi: 10.1371/journal.pone.0291029 (PMC10521995; doi:10.1371/journal.pone.0291029)
Supplement: S17 Fig — Size of each node represents level of pathway enrichment. Fold change represents logFC values of each gene compared to the WT. A. Downregulated pathways. B. Upregulated pathways. (PDF) [file pone.0291029.s017.pdf]

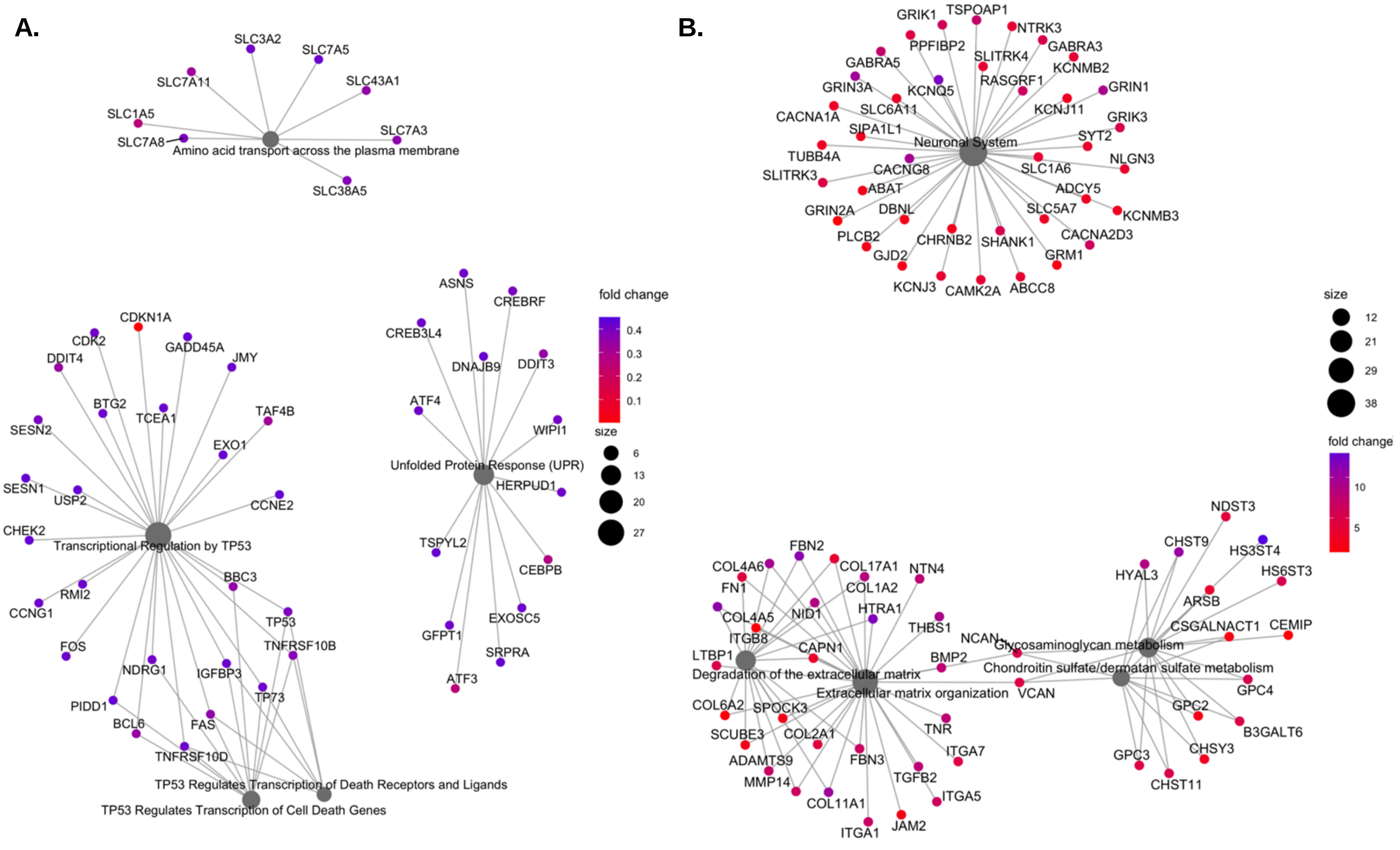

Supplementary Figure 17. Cnetplots of rs148726219-heterozygous iNeurons compared to the WT clone on day 23.

Size of each node represents level of pathway enrichment. Fold change represents logFC values of each gene compared to the WT. **A.** Downregulated pathways. **B.** Upregulated pathways.
