# Supplementary material for: Functional characterization of a single nucleotide polymorphism associated with Alzheimer’s disease in a hiPSC-based neuron model
Source: PLoS One. 2023 Sep 26;18(9):e0291029. doi: 10.1371/journal.pone.0291029 (PMC10521995; doi:10.1371/journal.pone.0291029)
Supplement: S18 Fig — (top) Single-frame images of day 45 iNs (WT 2A1, HOM 2H6, HET 2D2) transduced with AAV6-syn-GCaMP6f. (bottom) Representative raster plots visualizing neuronal circuit activity of each line. Individual lines show firing events of 50 iN cells per frame, imaged over 2000 frames (1 sec/frame) and lineup of individual lines indicates neuronal circuit formation. rs148726219-edited heterozygous hiPSC line (HET-2D2) shows enhanced functional circuit maturation compared to wildtype (WT-2A1) and homozygous (HOM-2H6) lines. (PDF) [file pone.0291029.s018.pdf]

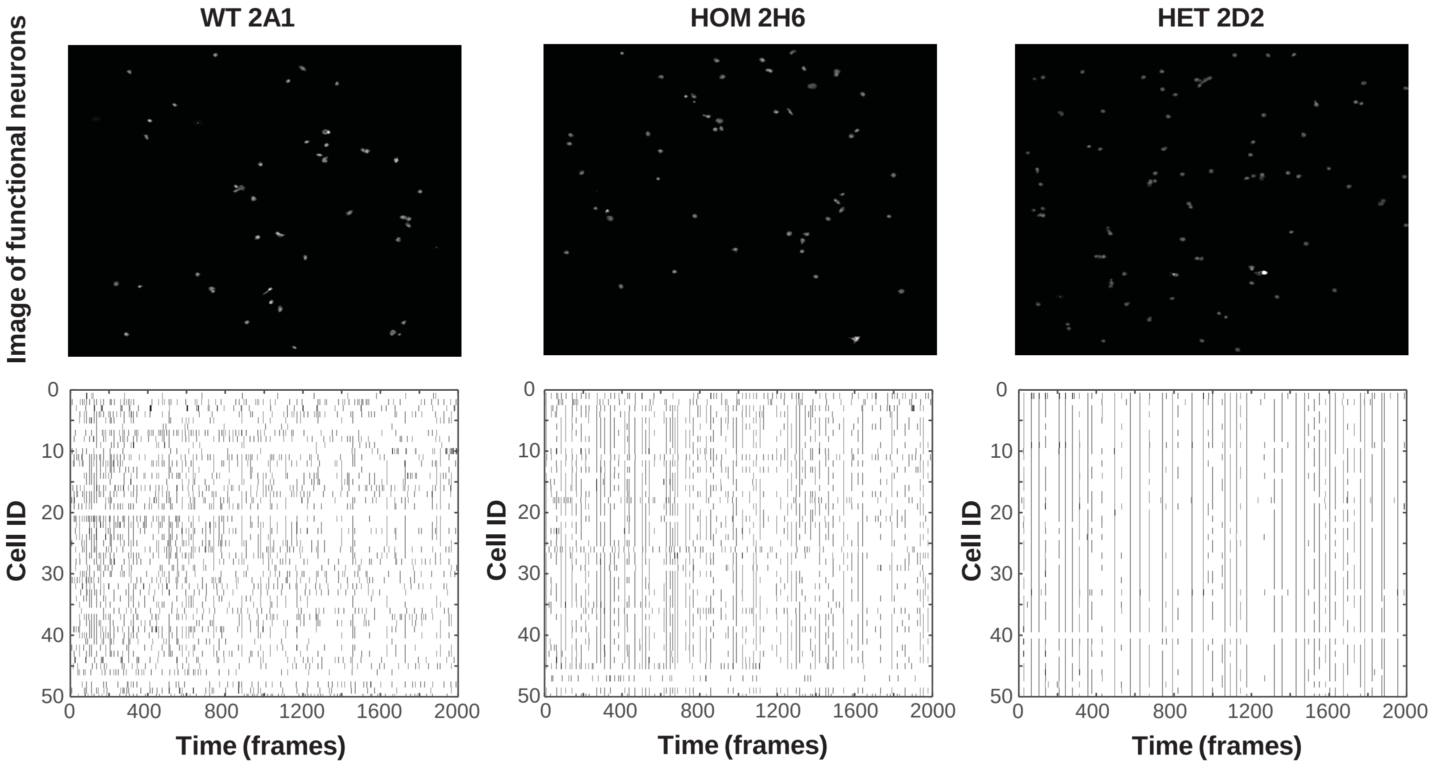

Supplementary Figure 18. Calcium signaling imaging of iN cells.
(top) Single-frame images of day 45 iNs (WT 2A1, HOM 2H6, HET 2D2) transduced with AAV6-syn-GCaMP6f. (bottom) Representative raster plots visualizing neuronal circuit activity of each line. Individual lines show firing events of 50 iN cells per frame, imaged over 2000 frames (1 sec/frame) and lineup of individual lines indicates neuronal circuit formation. rs148726219-edited heterozygous hiPSC line (HET-2D2) shows enhanced functional circuit maturation compared to wildtype (WT-2A1) and homozygous (HOM-2H6) lines.
