# Supplementary material for: Functional characterization of a single nucleotide polymorphism associated with Alzheimer’s disease in a hiPSC-based neuron model
Source: PLoS One. 2023 Sep 26;18(9):e0291029. doi: 10.1371/journal.pone.0291029 (PMC10521995; doi:10.1371/journal.pone.0291029)
Supplement: S19 Fig — Additional western blots of day 23 iNeuron lysates from independent hiPSC-iN differentiations of BIONi010-C-13 parental, WT-2A1, HET-2D2, HET-2G6, HOM-2B11, and HOM-2H6 clones. Blots were probed with an antibody against catalase and GAPDH as a loading control. The first experimental replicate is shown in Fig 6. A. Second experimental replicate. B. Third experimental replicate. (PDF) [file pone.0291029.s019.pdf]

A.

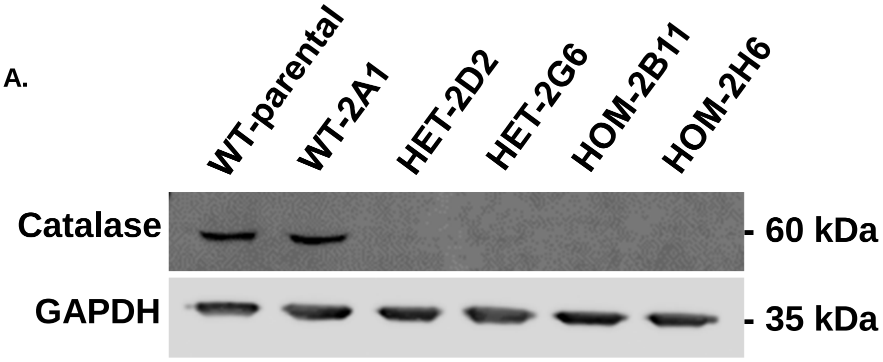

B.

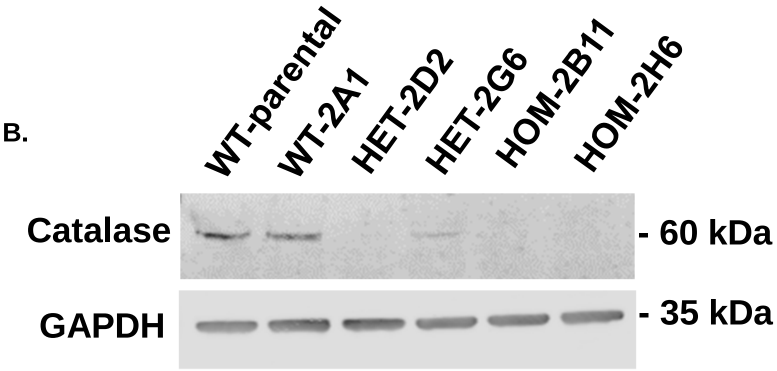

Supplementary Figure 19. Additional western blots for catalase expression used to perform quantification.
Additional western blots of day 23 iNeuron lysates from independent hiPSC-iN differentiations of BIONi010-C-13 parental, WT-2A1, HET-2D2, HET-2G6, HOM-2B11, and HOM-2H6 clones. Blots were probed with an antibody against catalase and GAPDH as a loading control. The first experimental replicate is shown in Figure 6. **A.** Second experimental replicate. **B.** Third experimental replicate.
