# Supplementary material for: Functional characterization of a single nucleotide polymorphism associated with Alzheimer’s disease in a hiPSC-based neuron model
Source: PLoS One. 2023 Sep 26;18(9):e0291029. doi: 10.1371/journal.pone.0291029 (PMC10521995; doi:10.1371/journal.pone.0291029)
Supplement: S20 Fig — Reads were aligned to hg38 using VarSeq. BIONi010-C-13 WT-parental, WT-2A1, HET-2D2, HET-2G6, HOM-2B11, and HOM-2H6 lines are shown as separate rows. Detected variants are shown as colored lines. No variants were identified in coding regions. Furthermore, no variants were detected that would explain CAT (top) or PCDHB5 (bottom) decreases in heterozygous and homozygous clones compared to parental or WT-2A1 clones. (PDF) [file pone.0291029.s020.pdf]

CAT

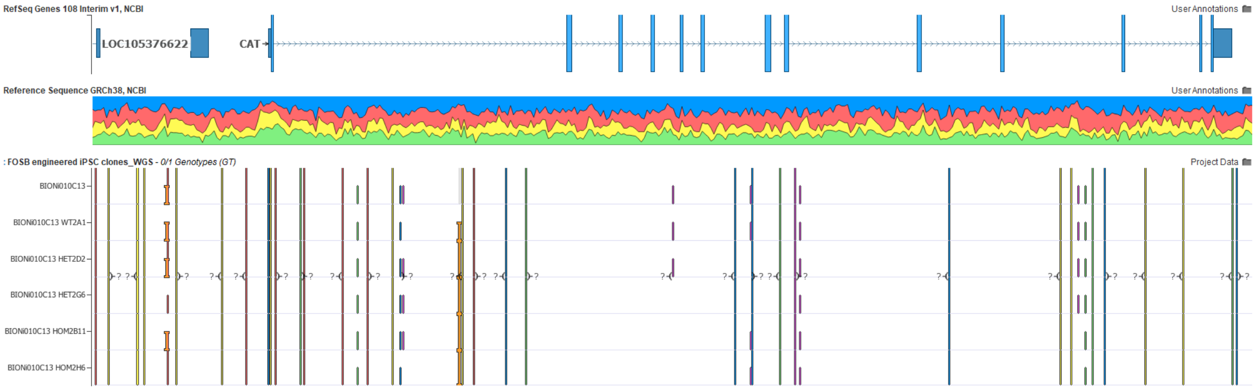

PCDHB5

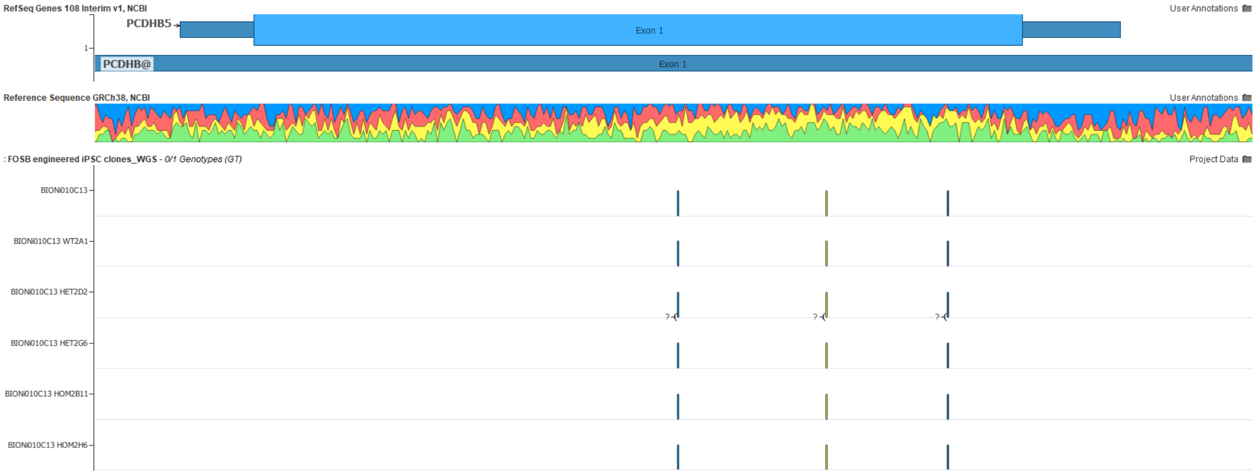

Supplementary Figure 20. WGS analysis of *CAT* and *PCDHB5* in rs148726219-edited hiPSC lines. Reads were aligned to hg38 using VarSeq. BIONi010-C-13 WT-parental, WT-2A1, HET-2D2, HET-2G6, HOM-2B11, and HOM-2H6 lines are shown as separate rows. Detected variants are shown as colored lines. No variants were identified in coding regions. Furthermore, no variants were detected that would explain *CAT* (top) or *PCDHB5* (bottom) decreases in heterozygous and homozygous clones compared to parental or WT-2A1 clones.
