# Supplementary material for: Functional characterization of a single nucleotide polymorphism associated with Alzheimer’s disease in a hiPSC-based neuron model
Source: PLoS One. 2023 Sep 26;18(9):e0291029. doi: 10.1371/journal.pone.0291029 (PMC10521995; doi:10.1371/journal.pone.0291029)
Supplement: S21 Fig — Log fold change (logFC) and FDR (adj.P.Val) values are shown for each gene in heterozygous clones (HET-2D2, HET-2G6) and homozygous clones (HOM-2B11, HOM-2H6) compared to the wild type clone (WT-2A1). Significant genes are denoted by colored boxes (blue = downregulated, orange = upregulated). “-” denotes genes not detected by RNA-seq. None of these genes were differentially expressed at any other time point. (PDF) [file pone.0291029.s021.pdf]

Regulators of *CAT*

DAY 23	HET vs. WT		HOM vs. WT	
Gene Symbol	logFC	adj.P.Val	logFC	adj.P.Val
CAT	-2.60373	2.66E-34	-9.62449	5.07E-42
NFE2L1	-0.62041	1.07E-14	-0.71272	2.36E-17
SP1	-0.40999	1.60E-09	-0.4904	9.46E-12
NFYA	-0.13709	0.004539	-0.14489	0.003027
XBP1	-0.9478	4.23E-11	-1.10658	2.38E-13
FOXO3	-0.67686	1.02E-12	-0.68755	7.47E-13
PPARG	-	-	-	-
POU2F1	-0.38858	1.73E-11	-0.37853	6.04E-11
CEBPB	-2.63889	3.63E-17	-3.04451	6.67E-18
PPARGC1A	-0.47268	9.28E-07	-0.60816	3.57E-09
SIRT1	-0.82156	7.77E-16	-0.78018	1.68E-14
FOXM1	0.955258	0.138725	1.815449	0.003618
FOXO1	-0.66644	0.215303	-0.87976	0.115817
WT1	-	-	-	-

AP-1 complex

DAY 23	HET vs. WT		HOM vs. WT	
Gene Symbol	logFC	adj.P.Val	logFC	adj.P.Val
FOS	-1.25933	3.56E-05	-1.49273	5.40E-06
FOSL1	-	-	-	-
FOSL2	-2.27759	3.84E-23	-2.58853	2.59E-24
FOSB	-	-	-	-
JUN	0.241349	0.044348	0.25418	0.034839
JUND	-0.86237	2.30E-15	-0.85626	5.28E-15
JUNB	-0.67921	0.000202	-0.54649	0.003384
JDP2	-	-	-	-
ATF1	-0.33302	0.012655	-0.19999	0.145409
ATF2	-0.37745	1.85E-07	-0.43862	3.66E-09
ATF3	-2.80948	4.07E-17	-3.39218	1.28E-18
ATF4	-1.26372	2.71E-15	-1.3492	1.81E-16
ATF5	-3.16154	5.73E-21	-3.39651	1.18E-21
ATF6	-0.41651	5.27E-16	-0.39753	6.17E-15
ATF6B	0.979421	1.68E-20	0.968078	5.55E-20
ATF7	-0.07818	0.166446	-0.12827	0.022784
ATF7IP2	0.127178	0.275378	0.083128	0.494855
ATF7IP	0.050849	0.447434	0.062624	0.34221
ATFX	-	-	-	-

Supplementary Figure 21. Expression of known regulators of catalase and members of the AP-1 complex identified from RNA-seq on day 23 of hiPSC-iNeuron differentiation.
Log fold change (logFC) and FDR (adj.P.Val) values are shown for each gene in heterozygous clones (HET-2D2, HET-2G6) and homozygous clones (HOM-2B11, HOM-2H6) compared to the wild type clone (WT-2A1). Significant genes are denoted by colored boxes (blue = downregulated, orange = upregulated). “-” denotes genes not detected by RNA-seq. None of these genes were differentially expressed at any other time point.
